# Harnessing metabolomics to better understand exercise‐mediated substrate metabolism

**DOI:** 10.1113/EP091127

**Published:** 2023-05-01

**Authors:** Colleen S. Deane, Jonathan R. Swann

**Affiliations:** ^1^ Human Development & Health, Faculty of Medicine University of Southampton, Southampton General Hospital Southampton UK

**Keywords:** amino acids, bioinformatics, exercise, glycogen, heat stress, metabolomics, protein

1

As global temperatures rise and major sporting competitions continue to diversify into warmer continents (e.g., FIFA World Cup Qatar 2022), intricate understanding of how the body responds to exercise in the heat is a ‘hot’ topic. Exercising in temperatures exceeding 30°C is associated with impaired endurance exercise capacity and altered substrate metabolism. This is characterised by increased reliance on carbohydrate utilisation and decreased lipid oxidation based on higher respiratory exchange ratio values and the analysis of a handful of traditional metabolites such as glucose and lactate. This understanding has underpinned the carbohydrate‐centric nutritional recommendations for exercising in the heat, which advocates a high carbohydrate intake pre‐, during and post‐exercise to support the increased carbohydrate demands and post‐exercise glycogen resynthesis (Burke, 2001).

Recent lines of evidence capitalising on advances in nuclear magnetic resonance (NMR) spectroscopy and mass spectrometry have provided a broader biochemical window into the metabolic response to exercise. This approach, known as metabolomics, aims to holistically measure the low molecular mass metabolites in a sample or system. This can include biofluids (e.g., urine, blood, saliva) and tissue samples (e.g., muscle biopsies), providing systemic and local information, respectively. The observed metabolome includes molecules that act as substrates, intermediates, reactants, products of enzyme‐mediated reactions and signalling molecules, and provides a high‐resolution overview of the metabolic state of the biological system being studied (Sakaguchi et al., 2019). In the context of exercise, metabolomic analysis revealed that prolonged endurance exercise lasting over 2 h altered over 300 metabolites in the blood (Nieman et al., 2013). This highlights the complexity of the metabolic response to exercise, which thus cannot be fully characterised by the analysis of a handful of metabolites. Importantly, the metabolome is specific to the individual, being a product of intrinsic factors such as the genome and its expression status, and extrinsic pressures such as dietary intake, physical activity and psychosocial stress. Hence, studying the metabolome can improve our understanding of inter‐individual variation in response to exercise and nutrition. While the application of metabolomics within exercise physiology is still in its relative infancy (Bennett et al., 2023), particularly in regard to environmental exercise physiology, improved understanding of the global biomolecular response to exercise is anticipated to support a step‐change in sporting nutritional guidelines.

In this issue of *Experimental Physiology*, Bennett and colleagues address part of this research gap by characterising the systemic metabolic response to exercise during environmental heat stress (Bennett et al., 2023). The authors performed untargeted metabolomics, using ^1^H‐NMR spectroscopy, on venous blood samples obtained from 23 male triathletes (${\dot{V}}_{O_{2}\mathrm{peak}}$ > 60 mlkgmin^−1^) pre‐ and post‐30 min cycling capacity test in hot (35°C) and temperate (21°C) conditions. Physiological variables changed as expected, with mean power output lower and mean heart rate, peak core temperature, core temperature change and peak rate of perceived exertion higher in hot versus temperate conditions. At the metabolome level, 35 unique plasma metabolites were measured, and a principal components analysis (PCA) model identified a distinct exercise‐associated metabolic response. Univariate analysis revealed 11 metabolites changed following exercise in temperate conditions, including amino acids and molecules involved in energy metabolism, with only an additional three metabolites altered in response to exercise in the heat. This suggests that endurance exercise evokes a similar systemic metabolic response in both temperate and hot conditions. However, when metabolites were expressed as change in relative abundance from pre‐ to post‐exercise within subjects, multivariate PCA and partial least squares‐discriminant analysis models identified distinct clustering between environmental conditions with 10 metabolites driving this distinction. In hot conditions, there was an increase in glycolytic metabolite abundance (e.g., glucose), consistent with prior reports, and alterations to the circulating amino acids. For example, alanine increased during exercise and was augmented in the heat relative to temperate conditions. Alterations in leucine, a vital muscle protein synthesis precursor, was also detected. These novel findings pertaining to amino acids call for more research into the impacts of heat stress on protein metabolism, both systemically (i.e., plasma) and locally (e.g., muscle). Such research could have important implications for nutritional recommendations surrounding exercise in the heat, which currently overlook the importance of protein intake relative to carbohydrates and could thus compromise muscle recovery. Whether temperature‐specific differences occur in major metabolic organs, such as skeletal muscle, remains to be determined and should be a focus of future investigations. It is also noteworthy that metabolites are highly labile, and capturing a single post‐exercise time point provides only a snapshot of the biological system as it returns to biochemical homeostasis. Greater temporality (e.g., multiple during and post‐exercise time points) will provide more comprehensive insights into heat‐related metabolic demands during exercise and recovery and should be carefully considered in future study designs (Castro et al., 2020).

This research by Bennet et al., provides an example of how metabolomics can be effectively applied within the exercise physiology context and can provide informative insight. Considering the increasing application of metabolomics within the field of exercise physiology (e.g., Heaney et al., 2019; Khoramipour et al., 2022; Sakaguchi et al., 2019; Schranner et al., 2020) and other relevant areas such as ageing (Wilkinson et al., 2020) and muscle deconditioning (Alldritt et al., 2021), we and others (Castro et al., 2020) suggest that now is the time for experts from all relevant fields to come together to discuss and establish standardisation guidelines and recommendations that aim to maximise the quality and robustness of future metabolomic studies and datasets. This will become even more important as platforms for metabolic phenotyping become more accessible and affordable, and nutrition and physical activity recommendations become tailored to the individual's metabolome. This personalised approach is anticipated to be a major component of exercise physiology in the 21st century, and its benefits are expected to extend beyond sports science and into many clinical settings.

## AUTHOR CONTRIBUTIONS

Colleen S. Deane conceived the idea. Colleen S. Deane and Jonathan R. Swann wrote the manuscript. Colleen S. Deane and Jonathan R. Swann approved the final version submitted for publication and agree to be accountable for all aspects of the work in ensuring that questions related to the accuracy or integrity of any part of the work are appropriately investigated and resolved. Both persons designated as authors qualify for authorship, and all those who qualify for authorship are listed.

## CONFLICT OF INTEREST

The authors declare no conflicts of interest.

## FUNDING INFORMATION

No funding was provided for this work.
